# Postmodernism and Education in Nursing Science: The Case of Clinical Skills

**DOI:** 10.1111/nup.70070

**Published:** 2026-02-23

**Authors:** Elissavet Nikolaou, Thalia Bellali, Eugenia Minasidou, Theodora Kafkia

**Affiliations:** ^1^ Department of Nursing International Hellenic University Thessaloniki Greece

**Keywords:** clinical skills, nursing, postmodernism, theory

## Abstract

During the development of theories that took place in the human and social sciences during the second half of the 20th century, when the current of postmodernism was particularly prevalent among French thinkers (French Theory), the human body preoccupied philosophy and sociology as a social construction in relation to other parameters such as the question of power and knowledge. The purpose of this study is to highlight the main aspects of the relevant interventions according to the pioneering conception of Michel Foucault, and the criticisms made by persons with opposing philosophical and sociological views on scientific questions, taking as a case study the medical and nursing care in the clinical field. One of the parameters that was affected is the way in which nursing education is influenced by the postmodern approach in the context of the clinical field such as operating theatre (OT), intensive care units, wards and the emergency department. One question is formed regarding the power of people in charge of nursing education, with the resulting discipline and the ability to produce knowledge, the way knowledge is preserved and the power structures within which nursing education is provided. The observation of nursing consciousness from the theoretical perspective of postmodernism gives rise to certain hypotheses regarding the contribution to maintaining knowledge and the implementation of good practices in the clinical environment. Similarly, the development of skills raises the question of contribution to the maintenance of a nurse's knowledge and empowering him/her to provide quality healthcare.

## 1 Introduction: The Postmodern Assault on Science

Looking at science and truth from within a postmodern lens, evidence‐based research can be seen as a social construction affected by culture and power. What is considered *true* is relative and a product of the dominant group's norms and interests (Kuntz 2012). In the field of nursing, postmodernism influences clinical skills education with a greater focus on context‐specific knowledge, communication and ethics, emphasizing patient values and cultural diversity.

## 2 Science and the Question of Truth

### 2.1 Postmodernism

Postmodernism considers representation as a mediated process, in which the real element and the represented element are subject to the observer's judgement. Mediation in the representational process proved to be an intractable problem in social research. Difficulties of decoding a particular representation relate to the study of the available data. Social sciences have developed tools and techniques to test for any errors and reveal the *hidden* dimensions of mental representation, the *noise* during the transmission of information (Mudrik et al. 2025).

Poststructuralism challenges scientific knowledge. According to postmodernism, science, interwoven with rationalism—from the Enlightenment to modern times—does not reveal the truth. It is not possible to know the real thing. The framework within which poststructuralism develops includes globalization, new technologies, the end of certainties in various theories of science, political upheavals, multiculturalism, consumer society, the emergence of hybrid forms in the various fields of culture and the shift from the public to the collective. Poststructuralism opposes rationalism, science, power and the anthropocentric view of the world and advocates the conjugation of meaning and existence. Scientific knowledge is not considered to be *superior* to experiential knowledge. Society exercises power through the *grand narratives* (representations constituted into wholes), and it is therefore advisable for the observer to pass on to *little narratives* (self‐contained representations separate from each other) (Papadopoulos 2022). Postmodernism raises the question of whether science exists. The separation of event and fact in postmodern approaches to science prepares the scientist to accept that the representation of the real element is impossible (Holtz 2020).

An event is something that has taken place, while a fact is the representation of the event by the scientist. According to postmodernism, the scientist refers to facts, since the study of events is mediated by representations. The scientist constructs the fact and therefore moves away from the event, from reality, reporting to us a narrative, that is, an unreal situation of the event. Postmodernism, opposing grand narratives focuses on the problem of language by giving it *absolute power* over the question of acknowledging the event (Connor 1999). Poststructuralists give priority to the signifier, the word, over the signified, the concept (linguistic turn), arguing that the signified is not limited to the meaning it signifies, but participates in a broader myth‐narrative so that the reader of the sign understands, along with the specific signified, its wider context (Deetz 2003). Those signified as part of a wider myth are responsible for perpetuating man's suffering, turning him/her into a passive receiver of the messages transmitted through myth by numerous power centres. Postmodernists, by proposing the *little narratives*, oppose textuality. Using the method of deconstruction, they attempt to bring to light the vulnerabilities of the text, its contradictions and the authoritarian moods of the *grand narrative* (Carr and Zanetti 2001).

At the opposite pole, a critical approach to postmodernism finds that reality has been identified with narrative. The view that people live in a world of copies, perceiving these elements and not reality, denies the whole edifice of civilization, any mental effort of the individual to understand his/her environment and improve his/her living conditions. Postmodernism's view of the undifferentiated identity of *grand narratives* is, according to its opponents, simplistic. By concentrating on the issue of power, postmodernism underestimates the role of other factors in cultural and individual life (Wilson 2022). By linking scientific knowledge, regardless of its ideological origin, with dominant repressive authoritarian schemes, it recommends a pluralism of minor powers that may lead to a position of *anything goes* or *whatever it is, it is okay* (Lyotard 1997).

By way of conclusion, postmodernism's scepticism towards grand narratives leads to a rejection of established rules and a focus on subjectivity, exploring its deeper implications, such as the social construction of reality and the deconstruction of assumptions.

### 2.2 The *Little Narratives* and the Question of Truth

Modernism and structuralism relate to positivism, whereas postmodernism and post‐structuralism correspond with phenomenology, and critical theory constructivism (Yousef 2017). For modernism, the threat to conventional order comes where conceptual categories change at a pace different from linguistic ones. In a situation where the pattern that language and thinking impose on experience necessitates revision. It occurs when new experiences contradict existing frameworks, forcing an adaptation in how reality is perceived or conceptualized (Howell 2013).

Poststructuralism covers several associated analyses of the relationship between power and knowledge, which have in common the view that knowledge is always contextual, partial and fragmentary, but also is never neutral and shapes the power relations between individuals or groupings. Poststructuralists criticize *grand* theories or systems of thought that make claims to uncover truth, including science. This relationship between power and knowledge can have consequences for subjectivity and identity. Postmodernism, closely associated with poststructuralism, seeks to undermine the *grand narratives* of modernist social organization and domination (Fox 2014).

The debate about *little narratives* leads to the question of truth. The positivist way of thinking, in an effort to capture the truth, seeks to reach conclusions without the mediation of ideology. In seeking truth, the rational technocratic way of thinking uses the perspective that suggests that social context should be considered. To discover the truth of the fact, it puts the content of the fact at the core of a reflection, according to which the social environment is not ignored. Instead, *little narratives* replace *grand narratives* that claim universal truth (Lyotard 1984). The question of truth becomes contextual; instead of a single truth, there are multiple, fragmented and competing *little narratives* that lack a claim to universal validity (Baier 2024). Within postmodernism, *truth* is not a single, discoverable fact, but what is accepted as true within a given narrative. There is no single criterion to judge the truth of all narratives; instead, different *language games* have different criteria for evaluation (Pukhaev 2022). The move to little narratives is a way to resist the totalitarian potential of metanarratives, which marginalize other experiences (Lyotard 1984).

As a last word, Wittgenstein's *language games* reject the idea that language is separate from reality. *Language games* are fragmented, with each having its own criteria for truth. The meaning of the word depends on the *language game* within which it is being used.

### 2.3 The Emerge of French Theory, and Foucault

Post‐war thought in France brought back the ideological and philosophical question of whether people's consciousness creates their existence, or whether their existence within society creates their consciousness (Canales 2016).

The emergence of French thought in Anglo‐Saxon literature sparked related conflicts over the question of reality (Cusset 2008) and established a different understanding of the mainstream theory and its intellectual bases (Birns 2014). The works translated from French were constructed in Anglo‐Saxon thought under the term *French Theory* (Gane 2003). In the meeting between Chomsky and Foucault, during which there was a confrontation between conceptions of the innate‐biological or acquired‐cultural nature of man, the common thread, the struggle for social justice (Wilkin 1999), extended to questions of epistemology, history, freedom and politics (Chomsky and Foucault 2006). At issue for Foucault were the institutions, which possess hidden power and serve to perpetuate the power of the dominant social class. According to Foucault, it was necessary to criticize the functioning of institutions and to expose the invisible violence they exert and the resulting inequalities in advanced societies (Chomsky and Foucault 2011).

Foucault, a post‐structuralist who rejected modernity, distinguished himself as a theorist in defining disciplinary society and disciplinary institutions. At a move from the *grand narrative* of the capitalist system (Beaulieu 2005), Foucault believed that the alternation of systems of maintaining order corresponded with the way in which the control of European society developed. As far as surveillance was concerned, within the population, there had been a revolutionary reshaping of society. The past anatomical approach to surveillance had been replaced by the social approach, with the recognition of the social dimension of the body. Surveillance was no longer practiced on physical bodies, but on thinking people. Disciplinary power emerged, according to which bodies were regulated by a power that was not inflexible. While, in the past, surveillance and punishment had taken the form of the exercise of power by rulers, in the new system of order, surveillance and punishment were now dependent on social, cultural and historical context. They constituted social constructions (Foucault 2012). Surveillance, and at the same time the enforcement (Foucault 2002), concerned persons for whom the purpose was remedial, since the power was not seeking to confirm its validity. Foucault saw power as a productive element, as an element of creation of reality. Power produces reality; it produces fields of objects and truths.

The disciplinary power, the power to normalize, had preconditions. It was becoming necessary to enrich the relationship between knowledge and power. Those in power had a fund of knowledge (Verdin et al. 2016), with which they devised normalization techniques (Foucault 2004).

Power and knowledge are not independent but feed off each other. Power makes knowledge possible and vice versa. Knowledge provides a channel for a group's claim to authority and control. The field of studies and the knowledge have a dual origin; it is a set of practices by which individuals become subjects of power, and also a grouping of individuals for academic or professional pursuits. Cognitive skills are not identified with institutions or mechanisms; they constitute a way of exercising power, which includes techniques and goals; it is a technology of power. Power technologies are disciplinary techniques that act on the body and produce the individual as an object. The techniques of power are combined to produce obedient bodies in such a way that they can be manipulated, transformed or excluded by those in authority. The techniques imposed on the body for its subjugation are observation, normalization and control. Observation supports a hierarchical power structure. Normalization is the practice in which individuals comply with standards. The techniques of observation and normalization further constitute the control, a disciplinary technique in which a person's proficiency in knowledge and skills is assessed. The productive capacity of power increased when surveillance and discipline technologies, which were used on individuals who were subject to authority, produced new knowledge, creating diverse disciplines in the humanities and social sciences.

The adoption of Foucault's perspective led to various forms of subjectivity. Power as a concept constructed outside the subject, with a function outside the subject, reduced individuals to the level of passive receivers, under constant surveillance, without the will to act autonomously, deprived of the ability to form an individual identity and prey to social control through examination and normalization. The techniques of authority, which make the body passive and obedient, also exercise power through mental functions and man's emotional and moral world, in the form of internal submission. However, the active subject tends to be constructed when exposed to a rigorous self‐examination. The *gaze* of the holder of power compels individuals to face their conscience and, albeit passively and obediently, to enter into an exchange with the power exercised over them, and to exercise power themselves. Subjects have the ability to construct themselves and regulate their own behaviour. Faced with the norms of behaviour currently in force in society and the control of practices through prohibitions, the individual can construct a behaviour governed by the cultivation of the self and make morality a matter of autonomy.

The ideological confrontation between modernity and postmodernists fuelled extensive discussions in modernist circles, among philosophers who attempted to understand the evolution of modern society and the behaviour of individuals. In a case, Habermas, confronted with Foucault's conceptions of power, advocated the creation of a political philosophy based on the recognition of people's communicative abilities and communicative rationality (Allen 2009), challenging common assumptions about the nature of science and its active part in the evolution of society (Kelly 1994). The discussion expanded to the question of the authority of science at a time when irrational forms of scientific scepticism were coming into contact with renewed forms of an obscurantist belief in the power of scientific knowledge (Mcintyre 2021).

In conclusion, Foucault's approaches to power reveal that science is not a neutral pursuit of objective truth, but is instead intertwined with power/knowledge systems that define what is knowable and true, shaping how individuals and societies are understood and controlled. These systems create ‘truth’ by producing specific discourses, categorizing individuals and using scientific knowledge to govern behaviour through techniques like medicalization and discipline (Guizzo 2021). Science, from this perspective, becomes a tool for shaping society, rather than an objective reflection of reality.

## 3 Health Sciences at the Center of Theoretical Debates

Health, a key field of discussion among theorists (White 2016), attracted prominent philosophers and sociologists who formed the vanguard of the postmodern movement. The views of modernists, notably Hans‐Georg Gadamer's philosophical position in 1993 on medical interventions and treatment of disease, on health, life and death (Dallmayr 2000), opposed Foucault's and other postmodern thinkers' views supporting the complicity of knowledge and power in the question of examining the patient's body and objectifying it.

Foucault's theoretical approaches defined the confinement of the patient in the hospital as a normalizing act. A characteristic of modern medicine and healthcare and of its related concepts is that patients become a group measurable, further manageable, a key aspect of the development of disciplinary power, emphasizing standardized, evidence‐based and data‐driven approaches (Broadbent 2019). Foucault's conception of medicine, of the medical institution, and of research structures for the production of medical knowledge about the human body was that, during the historical development of the university hospital, power deriving from the *medical gaze* had prevailed. The physician's practice tended towards the objectification of the patient's body as a separate and independent element of personal identity, in contrast to the earlier historical period in which it was believed that the human body is the person. In the treatment of disease, the structures of the hospital—both spiritual and material—made it possible to examine the human body, but clinical practice constituted part of the socioeconomic interests of power. When the patient's body entered the field of medicine, it also entered the field of power, within which the patient could be manipulated by the medical gaze (Misselbrook 2013).

In modern times, events have established the *grand narrative* of scientific discourse that has presented health scientists as individuals demonstrating excellent acumen who would fight disease effectively. This biological reductionism empowered physicians to apply the medical gaze to the patient's body, with an undeniable medical understanding of the patient's condition. The physician's ability to discover truth constituted the cultural perception of the medical gaze. Foucault's view of the hospital was not an observation based on rationality aimed at delineating the elements that made up the past, but a move that brought about a reversal in the mental structures for the production of knowledge and in the basic concepts and experimental practices of this discipline, with the restoration of clinical medicine as a new way of thinking about the body and disease, disease and medicine. This paradigm shift (according to Thomas Kuhn's view) replaced old scientific concepts with new ones. The hospital was founded on the new medical practice of verifiable observation, scientifically more accurate than the old empirical form of medical practice. A medical diagnosis was correct in the sense of its alignment with the mainstream mode of thinking in which the corresponding medical knowledge was seen as approaching reality (Suijker 2023).

Postmodernism denied the idea of a unique, universal truth about health, which was based—until the 20th century, in modern society—on rational science; it also rejected the existence of a single reality, and excluded the unique description of the past by emphasizing the coexistence of multiple realities. Postmodernism rejects the narrative validated by the mainstream of health sciences, with the acceptance of many little narratives, which do not focus on the important events that mark health but on the simple aspects of everyday life. Postmodernism emphasizes on discontinuity and diversity. The hierarchies of knowledge are nullified, and the ideas of a member of the social base, who happens to be a non‐specialist, are treated as having equivalent validity to the assessments expressed by the medical specialist, the select cultured part of society. Social construction denies the existence of a truth and the possibility of finding a single valid assessment of the body and diseases. It rejects the traditional narrative of medicine, which records a process of progress that has led to a sound knowledge of disease. It takes an eclectic approach: it presents a series of inconsistent arguments and does not attempt to provide a unified record of knowledge (Nettleton 2006).

The future theoretical development of health sciences is a matter of concern for both the medical and nursing sectors. Especially for nursing's scientific thinking, in the midst of the new post‐humanist theoretical currents that are developing in the humanities and social sciences, new horizons breaking away from the humanism of the Western world are sought in order to view nursing from a different perspective and further contribute to the understanding of nursing practice. This reflection extends beyond nursing science into the realm of social thinking and challenges the definition of health theory. The generally accepted, *legitimized* definition of social health theory, the approach that presents a systematic view of a health‐related phenomenon—one that is useful for describing, explaining, predicting or controlling it, is being challenged by the postmodern view. According to this view, social theory is considered an art, not always a science, whereby a scholar can ask the right questions about a health issue with the possibility of being at odds with the mainstream that has already settled it (Petrovskaya 2022).

## 4 Nurses and Clinical Environment

Nurses' training for the acquisition of clinical skills in the hospital acquires a different substance when it is discussed in terms suggested by postmodernism. In the case of hand hygiene, nurses' practices, for example, assuming the role of stewardship towards the patient (Murphy 2009), confer prestige on their identity. In most fields, such as prevention of infections or the drug administration via subcutaneous injections, the correct application of hand hygiene creates conditions for nursing staff to exercise authority in all directions, including chief nurses and doctors, with a view of providing quality care and preventing infections within the hospital.

Nurses experience anxiety during their intensive effort to rescue the critically ill patient within the hospital environment, using their acquired knowledge and techniques or following doctors' instructions. Hospital emergency practices may often violate administrative procedures (admitting and recording the patient), with the immediate transfer of the patient to the accident and emergency shock room and the OT, where the staff's agonized efforts to keep him alive unfold (Piagnerelli et al. 2009).

In the practice of nursing science, the generally prevailing perception of nurses—a quite old health profession—keeps them to the level of a job oriented towards the technical aspects of practice, dominated by constraints outside the nurses' sphere of influence (Duan et al. 2024). In some cases, nursing is regarded as a profession devoid of scientific thinking and personal initiative, resembling to the work of a hospital's auxiliary staff (Mishra 2015). In the case of the OT, the perceived subordinate position of nurses stems from the creation of a distance between their duties within it and the duties of nurses working in a hospital ward. In the wards, nurses develop personal relationships with patients, particularly those who are hospitalized for long periods, thus ensuring a fruitful communication that greatly facilitates required nursing interventions (Kourakos et al. 2017).

A beneficial feeling of satisfaction, with a consequent self‐confidence, accompanies the nurse during his/her implementation of nursing interventions, in accordance with his/her knowledge and experience, which restores the patient's health and reduces their anxiety (Manomenidis et al. 2017).

Nursing work in the OT, on the other hand, drastically limits a nurse's communication with a patient to the initial arrangements that must be made before the anaesthesiologist proceeds with anaesthesia. The inability to communicate with the patient during surgery inhibits the development of a personal relationship with him/her. Within the OT, despite the existence of organizational and other restrictions, there is scope for nurses to construct their professional identity. A nurse can actively contribute to the surgery, utilizing his/her scientific knowledge and techniques. Experienced nurses are able to contribute to the different phases of a surgery and can actively participate in the diagnosis and treatment of the patient's problem. While, historically, in major operations, the most important member of the surgical team was the surgeon (primary contributor), the operating theatre nurse [OTN] has now ceased, during the development of his/her role, to be a low‐ranking subordinate with little role in the surgical process. In major operations and/or in transplants, he/she may assume the more important role of a contributor, holding hooks or cutting the stitches needed by the surgeon. In the case of an emergency major operation during the night, if the surgeon does not have a trainee surgeon to help him, a nurse may undertake to participate as the surgeon's second contributor. In routine operations, in fistula creation or a central venous catheter insertion, nurses can help as a second contributor (Grota et al. 2021).

In certain circumstances, such as war, nurses may intervene as a primary contributor. In one case, Christiaan Barnard, who is best‐known for his first heart transplant operation in 1967, said of his assistant, Hamilton Naki—a person who had only empirical knowledge in the health sciences—that he exhibited technical skills that were superior to his own, especially regarding suturing, and had such a good presence in the OT that he considered him to be a better surgeon than himself (Zühlke and Mayosi 2017).

An understanding of clinical nursing from the sociological perspective promotes an in‐depth approach to the specialized nursing practice. In the sense of subjectivity and power, nursing appears to undergo social construction as a branch of knowledge and to be subject to the imposition of discipline. In their actions, nurses play a role in the governance and social construction of their profession. In the case of the OT, some easily distinguishable elements are the management and regulation of space and time to ensure the sterility of the surgical environment, and the emergence of the ethical concept of surgical consciousness (Porter‐O'grady and Pappas 2025).

A consideration of the OTN based on Foucault's positions shifts the focus onto different analyses of the elements that constitute the nurse's role. Foucault's work does not serve as a guide to the nurse's intervention next to the surgeon, on a patient's behalf, in the OT. It is used to analyse OTN's practices and knowledge. It can highlight the knowledge and practices displayed at the microlevel of nursing in the OT that distinguish it from other areas of nursing and healthcare. It highlights the way in which the OTN constructs his/her specialized knowledge and its expression through undertaken tasks in the clinical environment. The adoption, as an analytical category, of Foucault's productive nature of power opens up different ways of interpreting the evolvement of nursing in the OT. Nursing practices, and obviously the corresponding knowledge, have developed through the productive nature of power, contributing to the establishment of the specific specialization of nursing, in the course of a process in which the nurse shapes his/her practice and others shape his/her practice. One of the productive aspects of power is related to the empowerment of nurses, and has the potential to change their practice, with positive results, the improvement in the quality of their professional work (Udod 2008).

In a historical flashback, the evolution of health science has reduced morbidity and mortality in populations. The clinical environment, a field of new health‐related conditions, has been modified so as to reflect advances in the implementation of an aseptic environment. In the case of OTNs, with their specialized knowledge, it is found that they have acquired new responsibilities. Perioperative nursing has become enveloped by knowledge and focused on the OT environment, in terms of the necessary standards. To create an aseptic environment, the nurse, during the preparation process, disinfects the hands and forearms with surgical scrub and wears a sterile surgical gown and gloves. Surgical antisepsis has been proven to reduce the risk of infection. While organizing the equipment, in the process of counting the instruments and other objects, the nurse ensures, in each surgery, that the same order of counting is preserved, whilst also recording directly on the counting sheet (operation report) all objects added during surgery (Goodman and Spry 2016).

In line with Foucault's decentralized nature of power, OTNs are faced with expressions of power that are activated to restrict surgical costs. Practices such as reductions in material supplies and rationalization in the amount of available stocks limit the available surgical equipment. OTNs, as employees, are subject to the administrators' authority and their efforts to control costs (Lasater 2014). Even though OTNs are aware of the improved technologies that are available to facilitate surgery and the benefit to patient care. Power is exercised by administrators, surgeons and surgical equipment companies. Surgical equipment companies present their products to OTNs during conferences and at the nurses' places of work so that they are prepared when participating in management decision‐making. Apart from the companies, surgeons have an opportunity to explain the advantages and disadvantages of those materials so that nurses can form their attitudes when participating in administrative processes (Riley and Manias 2002).

When power is chosen as an analytical tool, an investigation is required, starting at the micro‐level of society, to reveal how power is produced, how its mechanisms work and how the relationships of those involved in health structures are constructed.

Relations of power at the micro‐level, OT, distracts the observer from the epiphenomenon, the nursing hierarchy and the dominance over the lower levels of it, and reveals the mutual exchange of power and knowledge (Ylitörmänen et al. 2023), the disciplinary self‐persuasion and the techniques used by nurses (Prinsloo 2024).

In the case of maintaining the integrity of the sterile field during a surgery, an inexperienced nurse, under the rule of technologies of discipline (Lawlor and Nale 2014), undergoes double scrubbing under the supervision of senior nurses who are capable of providing psychological support and helping him/her to acquire the necessary skills. By means of the double scrubbing, surgical interns and trainees are now able to participate in the preparation of the OT, and to gain experience of how instruments and sterile materials are placed on the sterile table, making them to learn the correct order in which the instruments should be given to the surgeon so that they do not have to change positions. At the same time, if a trainee doctor remains slow in his/her movements, an experienced nurse can intervene and respond quickly to the surgeon's tools and materials requirements. The above intervention constitutes a reprimand to the trainee, and a form of minor punishment that reinforces normalization through hierarchical observation and control (Riley and Manias 2002). In future operations, the trainee will be able to take over the handling of the instruments without the support of another experienced person. He/she will be able to arrange, within an acceptable time limit, the tools and materials on the sterile table, to keep them in order and give them to the surgeon skilfully, anticipating the surgeon's needs or meet them immediately.

The disciplinary technologies of nursing in the OT ultimately appear to be ambiguous, with, on the one hand, the production of knowledge about this nursing domain and, on the other, the regulation and control of its functions (Riley and Manias 2002). The investigation of disciplinary techniques pertaining to OT nursing permits an examination of how authority is constituted to formulate and manage discipline. The nurses' self‐discipline and management of OT nursing is clearly demonstrated through the elucidation of how power operates, which, through normalization, controls the individuals' behaviour (St‐Pierre and Holmes 2008).

By choosing subjectivity as an analytical tool, subjects—who are constructed through their ability to contribute to their personal behaviour—present an ethical picture, which is evident in OT nursing in the form of surgical consciousness (Riley and Manias 2002), which helps to maintain a sterile field during surgical procedures.

Members of the ‘sterile’ group are encouraged to make a self‐examination of the technique they have used in a particular procedure through an internal examination of consciousness. Accidental violations of the technique that may contaminate the sterile field, such as contact with a non‐sterile object, are reported by the individual team member, although others may not have noticed the breach. Violations of the sterile process that have been observed by other team members are also reported through the clinical gaze, a general principle of surveillance, however, influenced by the authority of the other team members. OT nursing trainees feel powerless before an experienced nurse and are reluctant to report the violation, fearing criticism. A subject's position that an OTN adopts may affect the integrity of the sterile surgical field. Professional guidelines regarding aseptic technique and OT nursing (Lewis et al. 2014) provide ethical codes for the specific nursing specialization. At the individual level, the subjectivity of OTNs is structured by these historically developed codes. A nurse providing, to the rest of the team, information about any aseptic technique violations is a scientist that knows the correct aseptic technique and, at the same time, is an ethical nurse who respects the codes of the OT. On a social and cultural level, reporting violations of aseptic technique forms part of a nurse's moral and professional obligations and plays a part in constructing the discipline of OT nursing. Self‐discipline, for example, in reporting violations of aseptic technique, may act as a defensive barrier to mastery techniques, such as hierarchical control, as a nurse is accountable to himself/herself and finds personal moral satisfaction in adhering to the code of conduct (Riley and Manias 2002). The result is a certain freedom in the nurse's disposition to act and define himself/herself as a subject. OTNs, moving into a field of knowledge and practice, determine the parameters through which they may be freed from the influence of other professional groups and realize, in OT nursing, the effect of knowledge on power.

Beyond the areas of power and subjectivity, which are offered for discussion, the question of practice needs to be analysed. In OT, discipline shapes the professional activities of nursing practices by imposing defined modes of behaviour. The study of related material and the practices surrounding OT nursing provide a body of knowledge that can be applied in this field. Regulation of movement in a sterile area, organization of the stock, preparation of the equipment, time management and activation of surgical consciousness are all aspects of the discipline that contribute to the precision required to safely carry out surgeries. These disciplined practices, as well as the knowledge that shapes them, distinguish OT nursing from the mainstream currents shaping the nursing profession. The specific nurses see discipline as an arbitrary factor that confines them to a position of submission to other health professionals. The power‐knowledge of OT nursing is underestimated by nurses themselves and the wider healthcare community (Riley and Manias 2002). An analysis of the practices undertaken by the specific nurses leads to an understanding of how they build the prestige of their own field of expertise.

Through questioning or developing a problem in the field of practices considered as prevalent, the effects of the exercise of power are offered for investigation, by clarifying the way of thinking of OTNs when positioning them against power relations. This analysis permits an investigation of the actions of OTNs and the effects of those actions on the behaviour of others.

In the case of OT nursing, its analysis may lead to an investigation of the conditions under which they act as individuals in order to regulate their own practice, and may also lead to the identification of the conditions under which they, as a group of qualified scientists, act to construct knowledge that provides information regarding the practice at a wider professional level. The advantage of this analysis is that it challenges the forms of domination that have historically shaped the prevailing images of this field of nursing specialization and offers an opportunity to transform everything relating to OTNs so as to improve individual normalization. By questioning the structures of the OT, nurses may become more aware of their contribution to the nursing profession. OTNs need to understand how they wield power through their activities and recognize that their roles go beyond traditional interpretations that present their specialism as an inferior one. On the other hand, when the context of OTNs' practices and knowledge is explored, the consequences of power relations are subject to question. OTNs can then become more instrumental in legitimizing and promoting their work, developing strategic plans to ensure an outcome that is more visible (Riley and Manias 2002). Such strategies may include improvements in the documentation of nursing activities and the development of procedures included in general nursing textbooks. The effect of such strategies would be to actively promote the roles that OTNs may play in decision‐making in the clinical setting.

An issue of particular importance is the support of social institutions whose structures are grounded in the health sciences, in terms of space and time. Spatial segregation is one of the mechanisms by which a group can exercise power, controlling access to knowledge (Musa 2025). In the case of OT, where spatial structures have helped in the past to maintain differences between genders, lower‐ranking positions are subject to supervision and control over knowledge, and also suffer from a lack of privacy, unlike other, higher‐ranking positions that enjoy privacy and control of space. The social space in the OT has no fixed boundaries, and is subject to extensions or maintenance of the existing space, with any changes depending on social power relations in terms of social class, gender or race (Riley and Manias 2002).

In the clinical setting, individual areas are graded in terms of the degree of freedom or restriction of access to them. Some offer free access to staff. In partially controlled areas, access is regulated according to relevant instructions. In the case of the OT, offices, corridors, stockrooms and OTs are places accessible only to doctors, nurses and technical staff. Nurses and technicians know the working practices, while surgeons are ignorant of them. In fully controlled areas, halls are strictly regulated, with a ban on the entry of unauthorized persons. The aseptic technique is protected, following inflexible rules, by ensuring the environment is kept sterile so that the surgery can be performed without risks. The regulation of the movement of medical and nursing staff provides different entry and exit points than those for patients. Access to patients' wards, anaesthesia and OTs is minimized. Staff and patients on the operating table wear, according to the instructions, sterile surgical clothing. The equipment (tools and other materials) is sterile, with provision for placing it separately from contaminated material. The organization of the OT, the deposit of tools and other materials in lockers, the provision for the supply of stock for each medical specialism and the sterilization process all correspond to knowledge that the OTN is required to acquire (AORN 2020).

OTNs' professional associations have formulated standards of good practice that regulate the use of space during a surgical procedure. During surgery, certain movements are mandatory for staff in order to avoid contamination of the sterile surgical team. Personnel previously assigned to the surgical antisepsis procedure must remain within the sterile field. When changing places, doctors and nurses are required to maintain a safe distance from each other and use seats only when surgery permits it. The medical sector also has guidelines in conjunction with nursing standards. In the case of ensuring the sterilization of the OT's environment, the nurses' exercise of power over doctors, if the latter refuse to follow his/her instructions, takes place not through violent means but by the invocation of approved standards of practice, which force them to comply. The standardized repetition of activities by doctors and nurses, carried out in an effort to minimize the risk of harm, is interrupted by arbitrary regulations set by the prevailing forces of power (AORN 2020).

In the case of space use in the OT, cases arise in which a different delimitation is imposed, with the argument that the controlled internal environment should be prevented from becoming contaminated by the uncontrolled external environment. The demarcation points, which act as an infection control mechanism by separating the OTs from the rest of the hospital, place the nursing staff employed within the controlled areas in a commanding position due to their distinct type of knowledge. In other cases, flexible forms of power manifest themselves, with the adoption of adjustments to the needs of health personnel and the lifting of bans on their access to indoor spaces such as the recovery room (Riley and Manias 2002).

With regard to time as a disciplinary technique of power, the concept of regulated time in the OT is evident in the actions of staff. Sterile tool trays are packed according to their intended use and in the order laid down in a predefined list. These are counted in a systematic way: first, the discarded materials (gauze and other packaging) are counted, and then those kept in the sterile set, and finally, the materials that remain in the room. These are practices that regulate the use of time and the discipline in OT nursing, and constitute a specialized area of knowledge specific to this practice (Riley and Manias 2002).

The regulation of time constitutes an area for power conflicts within the clinical environment, when the interests of individuals with different roles conflict with the way in which the space is organized. The time in the OTs is divided into regular alternating intervals for different groups of employees, in the morning, in the afternoon and at night. When a surgeon exceeds the specified surgical time, OT supervisors face the possibility of cancelling or rescheduling remaining surgeries by activating the relevant procedures provided by the regulations. In the decision‐making process, hospital managers, under the stress for their patients, must, in addition to ensuring cooperation between surgeons and nurses, also protect the interests of the organization. Surgeons and hospital managers have the ability to exercise authority by increasing the number of cases on an OT list, despite the doubts expressed by the nursing managers that surgeons will be unable to complete scheduled procedures within the allocated time. Nursing managers, when needed, exercise power by having the authority to organize and rearrange the surgical lists and all related activities to ensure adequate space, staff and equipment. When managing time in the OT, nurses believe that changes in scheduling that have safety implications, with insufficient time between surgeries and an increased speed of activities, may lead to staff exhaustion or equipment damage. Externally imposed changes, with a reduction in time between different surgeries due to an increased workload, lead experienced nurses to take decisions that involve assuming greater responsibility without their being covered by corresponding changes in their support structure and professional responsibilities (Riley and Manias 2002). Operating room nurses can, however, change the way in which time is used in the OT to construct their own practice. They have the potential to exercise authority by reorganizing the way in which time is used in OT and, consequently, to construct and regulate the practice of others.

In conclusion, random descriptions of nursing activities in various areas reveal aspects of their work that can be analysed in a Foucauldian framework. Disciplinary techniques of power are used to maintain control within the hospital setting. Nursing disciplinary self‐persuasion is linked to the techniques nurses use to regulate their own behaviour to align with nursing values and standards of practice. These techniques involve various strategies rooted in professional values. The mutual exchange of power and knowledge in nursing involves sharing control and information between nurses, patients and other healthcare professionals to create collaborative, patient‐centred care. Furthermore, Foucault's theories of power and knowledge illuminate how hierarchy shapes nursing. Power operates through knowledge, where the dominant discourse of the hospital hierarchy creates nursing knowledge, which then disciplines the behaviour of nurses.

## 5 Rethinking the Practice of Nurses in the Clinical Environment

The framework in which the perception of health scientists is driven by Foucault's approach can lead to a rethinking of nursing education in terms of acquisition of knowledge and skills and implementation of good practices, thus contributing to an improvement in their professional image in the clinical environment and a strengthening of their position in the health sector.

Foucault's theory of power highlights the role of nurses in their environment as they are involved in the power‐knowledge and knowledge‐power relationship. The nurse acquires an enhanced profile and therefore can contribute. During the nurses' training, the element of their enhanced identity is considered and emphasized in order to understand that their profession is multifaceted. They acquire additional knowledge, which is geared towards shaping their own power. When the trainee nurses enter the workplace, they already possess the infrastructure to take on the role of steward and protect the patient. It is important to point out to trainees that the knowledge elements with which they enter the hospital carry special weight. Trainees will understand that the knowledge they possess and their new training compose a framework of upgraded knowledge that contributes to the improvement of professional response to the patient with the aim of providing quality health services.

## Funding

The authors received no specific funding for this work.

## Ethics Statement

The authors have nothing to report.

## Conflicts of Interest

The authors declare no conflicts of interest.

## Data Availability

The authors have nothing to report.
